# Pharmacokinetic and bioequivalence of two formulations of Valsartan/Amlodipine Tablet(I) in healthy Chinese volunteers: A randomized, three-period, partially replicated crossover study

**DOI:** 10.1038/s41598-025-32576-9

**Published:** 2025-12-13

**Authors:** Yingying Mei, Xu Zuo, Tiandong Zhang, Ziyun Bu, Zhendan Xu

**Affiliations:** 1 Phase I Clinical Trial Research Laboratory, Xinxiang Central Hospital, No. 56 of Jinsui Street, Weibin District, Xinxiang, 453000 Henan Province China; 2 The Fourth Clinical College of Henan Medical University, Xinxiang, China

**Keywords:** Valsartan/Amlodipine Tablet(I), Hypertension, Bioequivalence, Pharmacokinetics, Safety, Diseases, Drug discovery, Medical research

## Abstract

Valsartan/amlodipine combination tablets can exert synergistic antihypertensive effects through different mechanisms, significantly lowering blood pressure while also addressing multiple risk factors and comorbidities in patients. This study designed a single-center, randomized, open-label, two-formulations, single-dose, three-cycle, three-sequence, partially repeated crossover controlled trial design. A total of 72 healthy Chinese volunteers participated in the fasting and fed trials, of whom 69 completed the trial process. This study employed a methodologically validated liquid chromatography-tandem mass spectrometry (LC-MS/MS) method to determine the concentrations of valsartan and amlodipine in plasma. Phoenix WinNonlin software (version 7.0) was used to calculate pharmacokinetic parameters using a non-compartmental model. Equivalence analysis was performed using the average bioequivalence (ABE) method and the reference-scaled average bioequivalence (RSABE) method. The trial results showed that the test drug was bioequivalent to the reference drug, and no suspected serious adverse reactions or serious adverse events occurred throughout the trial. The results of this study support the launch of the new generic Valsartan/Amlodipine Tablet(I) in China. As a representative antihypertensive fixed-dose combination (FDC), these methodological insights can be directly applied to clinical trials of similar drug products, thereby enhancing success rates while mitigating development risks.

**Clinical trial registration**: CTR20221889, (http://www.chinadrugtrials.org.cn/clinicaltrials.searchlist.dhtml.28/07/2022)

## Introduction

 China is currently facing the dual pressures of an aging population and the prevalence of metabolic risk factors. The incidence and mortality rates of cardiovascular disease (CVD) continue to rise, making it the leading cause of death among the Chinese population. A meta-analysis examined the trends in hypertension prevalence in China from 1959 to 2018, incorporating 18 studies involving over nine million Chinese hypertension patients. The results showed that the annual growth rate of hypertension prevalence in China was 0.29% prior to 2004, and after 2004, it increased to 2.45%, approximately eight times the annual growth rate prior to 2004^1^. Unlike secondary hypertension, which has a clear cause, the pathogenesis of primary hypertension remains unclear. It is a complex multifactorial disease involving both neurological and humoral factors, influenced by the interaction of genetic and environmental factors. The causes and pathogenesis vary among individuals^2,3^. The fundamental goal of hypertension treatment is to reduce the overall risk of cardiovascular, cerebrovascular, renal, and vascular complications and mortality. The primary benefit of treatment stems from the reduction in blood pressure itself. The main drugs used clinically to treat hypertension are calcium channel blockers (CCBs), angiotensin-converting enzyme inhibitors (ACEIs), angiotensin receptor blockers (ARBs), diuretics, and beta-blockers, as well as fixed-dose combination formulations composed of these drugs^4^.

Amlodipine, adihydropyridine CCB used to treat hypertension, reaches itspeak concentration between 6 and 8 h after oral dosing. Amlodipine is mostly absorbed, metabolized in the liver, and excreted in the urine. Valsartan, an ARB with selectivity for the type 1 receptor subtype, is predominantly eliminated via biliary excretion Maximal plasma concentrations occur 2–4 h after oral administration, and only approximately 20% of valsartan is metabolized^5^. Exforge^®^ is a combination tablet developed by Novartis in Switzerland that contains valsartan and amlodipine. Exforge^®^ was launched in the European Union in January 2007, in Switzerland in February of the same year, and in the United States in June. On July 23, 2008, it was approved by the US Food and Drug Administration (FDA) for use as a first-line treatment for hypertension. In 2009, the National Medical Products Administration (NMPA) approved the import and marketing of Valsartan/Amlodipine Tablet(I) (trade name: Exforge^®^) developed by Novartis of Switzerland. The approved dosage is 80/5 mg, for use in patients with primary hypertension whose blood pressure cannot be adequately controlled with monotherapy. The efficacy and safety of Exforge^®^ are widely recognized by clinicians and patients. In order to alleviate the financial burden on patients with hypertension, provide additional therapeutic options, and improve their quality of life, Hubei Wushi Pharmaceutical Co., Ltd. has developed a new generic tablet containing valsartan and amlodipine (80/5 mg). In adherence to the “Opinions of The General Office of the State Council on the Consistency Evaluation of the Quality and Efficacy of Generic Drugs” (No. 106, 2016) set by NMPA, it is necessary to conduct bioequivalence (BE) studies to ensure the consistency of our generic drug with the brand drug (https://www.gov.cn/zhengce/zhengceku/2016-03/05/content_5049364.htm, Last access data 30 Jul, 2025).

According to the Catalog of Generic Reference Preparations (Batch 10, https://www.nmpa.gov.cn/xxgk/ggtg/ypggtg/ypqtggtg/20171013175801889.html, Last access data 30 Jul, 2025), Valsartan/Amlodipine Tablet(I) (trade name: Exforge^®^, 80/5 mg) manufactured by Novartis Pharmaceutica S.A. were selected as reference product. In this study, blood samples were collected from healthy Chinese volunteers under fasting and fed conditions after administration of the test and reference drugs. The concentrations of valsartan and amlodipine in plasma were determined by a validated LC-MS/MS method. Pharmacokinetic (PK) parameters were calculated to evaluate whether the generic drug exhibited bioequivalence (BE) to the branded product. Additionally, the safety profiles of both the test and reference drugs were assessed in healthy volunteers.

Valsartan is considered a highly variable drug (HVD) because only approximately 20% is recovered as a metabolite, and cytochrome P450 isozymes are known to contribute little to its metabolism^6^. Moreover, valsartan was revealed to be a HVD, as the CV% of the C_max_ is > 30%^7^. As a representative antihypertensive FDC, the BE study of valsartan-amlodipine tablets not only provides evidence for the market authorization of its generic versions but also offers critical reference for clinical trial design, methodological optimization, and regulatory strategies for other drugs—particularly FDCs and highly variable drugs (HVDs).

## Methods

### Subjects

This study enrolled 72 healthy Chinese volunteers (36 fasting and 36 fed), comprising both male and female participants aged 18 to 65 years (inclusive) with balanced gender distribution. The inclusion criteria required a body mass index (BMI) ranging from 19 to 26 kg/m^2^ (inclusive of threshold values), with additional weight requirements of > 50 kg for male participants and > 45 kg for female participants. Physical examination, medical history, and clinical laboratory test results of the subjects should exhibit no clinically significant abnormalities. Prior to the trial, all participants were thoroughly informed of the study’s nature, significance, potential benefits, possible inconveniences, and associated risks. They voluntarily agreed to participate in this clinical trial, demonstrated the ability to communicate effectively with investigators, committed to complying with all study requirements, and provided written informed consent.

Patients with a history of orthostatic hypotension or those presenting with orthostatic hypotension during screening, as well as individuals with a history of angioedema, were excluded from the study. Other major exclusion criteria included: (1) Participation in other drug clinical trials within the past 3 months; (2) A history of vomiting, diarrhea, or any physiological condition that could interfere with trial results within 7 days prior to the study; (3) A history of specific allergies (e.g., asthma, urticaria, eczema) or hypersensitivity to any medication, food, or pollen, as well as known allergies to valsartan or amlodipine; (4) Blood loss or donation exceeding 400 mL within 3 months prior to the trial or intention to donate blood during the study period; (5) Excessive daily consumption of tea, coffee, and/or caffeinated beverages, alcohol use, or smoking within 3 months prior to the trial; (6) Pregnant or lactating women, or subjects (including male subjects) with plans for conception, sperm donation, or egg donation from 2 weeks before the trial until 3 months after the last dose administration; (7) Subjects who underwent surgery within 3 months before the trial, or plan to undergo surgery during the study period, as well as those who have undergone any surgery that may affect drug absorption, distribution, metabolism, or excretion; (8) Individuals with a history of asthma or epilepsy; (9) Use of any medications (prescription drugs, over-the-counter drugs, health supplements, or herbal medicines) within 14 days before the trial; (10) History of substance abuse; (11) Inability to tolerate venipuncture and/or a history of vasovagal syncope (needle or blood phobia); (12) Volunteers deemed by the investigator to have any condition that renders them unsuitable for participation in this trial.

### Study drugs

Test drug (T): Valsartan/Amlodipine Tablet(I) Specification: Each tablet contains valsartan 80 mg and amlodipine 5 mg; Batch No.: 220,301; Expiry date: February 2024; Content: valsartan 99.3%, amlodipine 99.3%; Manufacturer: Hubei Wushi Pharmaceutical Co., Ltd.

Reference drug (R): Valsartan/Amlodipine Tablet(I), Trad name: Exforge^®^; Specification: Each tablet contains valsartan 80 mg and amlodipine 5 mg; Batch No.: BUJ41; Expiry date: August 2023; Content: valsartan 100.0%, amlodipine 100.5%; Marketing authorization holder: Novartis Pharma Schweiz AG, packaged by Beijing Novartis Pharma Co., Ltd. Drugs were stored below 30 °C).

### Study design

This study was conducted at the Phase I Clinical Trial Research Unit of Xinxiang Central Hospital. The study protocol and amendments were approved by the Ethics Committee of Xinxiang Central Hospital (Approval Nos. 2021-039, 2022 − 249, and 2022 − 537). Registration for this study was completed on the “Drug Clinical Trial Registration and Information Disclosure Platform” (http://www.chinadrugtrials.org.cn/clinicaltrials.searchlist.dhtml,28/07/2022,CTR20221889). Throughout the study, all procedures were strictly performed in compliance with the ethical principles for medical research involving human subjects as outlined in the Declaration of Helsinki, the Good Clinical Practice (GCP) guidelines issued by NMPA, the International Council for Harmonisation of Technical Requirements for Pharmaceuticals for Human Use (ICH) guidelines, and relevant domestic laws and regulations.

According to previous studies^8,9^, valsartan exhibited substantial intra-subject variability, with intra-subject coefficients of variation (CV) for AUC_0 − t_, AUC_0−∞_, and C_max_ being 30.0%, 27.6%, and 31.2%, respectively, classifying it as a highly variable drug. In contrast, amlodipine demonstrated relatively low intra-subject variability. Therefore, the sample size estimation for this study was based on the intra-subject variability of valsartan. The sample size was calculated using the intra-subject CV of C_max_ (three-way crossover, table look-up method). Taking into account the potential influence of food on drug absorption as well as the anticipated dropout rate, it was determined that 36 healthy volunteers would be enrolled for both the fasting and fed trials. To assess the BE of HVDs, which have relatively short elimination half-lives, a replicate crossover design with three or four periods would be appropriate. Under the same conditions, a four-period replicate crossover design requires fewer participants than a three-period replicate crossover design^10^. Since it is considered ethical for healthy participants to participate in a short duration and low exposure to drugs, the probability of losing participants increases as the study duration becomes longer.We performed a 3-period reference replicate crossover study with 36 subjects. Statistically, the replication of administration within a subject improves the quality of the data and leads to definitive study results^11^.

The study was designed as a single-center, randomized, open-label, two-formulation, single-dose, three-period, three-sequence, partially replicated crossover bioequivalence trial conducted under fasting and fed conditions in accordance with relevant guidelines^12,13^. Volunteers were assigned screening numbers in chronological order of screening. On Day − 1 of the trial, each eligible volunteer received a randomization number generated by the Statistical Analysis System (SAS) (version 9.4) using 1:1:1 block randomization, arranged from the smallest to largest screening number. The randomization number served as the administration sequence number, with 12 subjects allocated to each group. This study was an open-label study. The analysis and testing personnel would use blinded analysis and would not know the randomization codes of the subjects or the dosage formulations administered in each week during the sample analysis process. Other personnel such as clinical investigators, data management and statistical analysis personnel were not blinded.

Following the latest “Technical Guidelines for Human Bioequivalence Studies of Generic Chemical Drugs Using Pharmacokinetic Parameters as Endpoint Indicators” issued by NMPA^14^, the washout period between study phases was set to be at least 7 times the terminal elimination half-life. Considering that the terminal elimination half-life of orally administered valsartan in healthy subjects is approximately 9 h and that of amlodipine ranges from 30 to 50 h, a 14-day washout period was implemented to ensure complete drug elimination prior to subsequent dosing.The research sequence, cleaning period, and medication status for each cycle are shown in Table 1.

**Table 1:** Drug administration sequence Chart

Group	First Cycle	Washout period	Second Cycle	Washout period	Third Cycle
	Fed/Fasting	14 Days	Fed/Fasting	14 Days	Fed/Fasting
A	T		R		R
B	R		T		R
C	R		R		T

### Study procedure

Volunteers were admitted to the Phase I clinical unit 2 days prior to the trial (Day − 2). On the day before drug administration, an overnight fast of at least 10 h was required (water intake was permitted during the fasting period). On the day of the trial (Day 1), baseline blood samples were collected in the morning. Subsequently, volunteers orally received either the test or reference drug (1 tablet per subject) with 240 mL of warm water under fasting conditions. Water intake was prohibited from 1 h before to 1 h after dosing (except for the water taken with medication). Food intake was restricted for 4 h post-dose, with standardized meals provided at approximately 4 and 10 h after administration. For the fed group, a high-fat breakfast was consumed 30 min before drug administration. The high-calorie, high-fat meal provided approximately 800–1000 kcal, with macronutrient contributions as follows: ~150 kcal from protein, ~ 250 kcal from carbohydrates, and 500–600 kcal from fat. Volunteers were instructed to avoid strenuous physical activity after dosing and to maintain an upright position for the first 2 h post-administration. During the hospitalization period, only specified foods were consumed at designated times, with no additional dietary intake permitted.

### Blood sampling

Blood samples were collected under fasting and fed conditions for PK analysis. In accordance with the “Technical Guidelines for Human Bioavailability and Bioequivalence Studies of Innovative Drugs”^15^, the blood sampling time points were determined based on the reported pharmacokinetic parameters of valsartan and amlodipine^16–18^. For each study period, blood samples were collected at the following time points: within 1 h before dosing, and at 0.5, 1, 1.5, 2, 2.5, 3, 3.33, 3.67, 4, 4.33, 4.67, 5, 5.5, 6, 7, 8, 10, 12, 24, 48, and 72 h after dosing, totaling 22 time points. Approximately 4 mL of venous blood was drawn from the upper limb and collected in EDTA-K_2_ (Ethylenediaminetetraacetic Acid Dipotassium Salt) anticoagulant vacuum tubes. To ensure sample stability, the blood collection tube should be gently inverted six times to thoroughly mix with the anticoagulant. The sample was then placed under yellow light at room temperature and subsequently centrifuged (centrifugation parameters: temperature set at 4 °C, relative centrifugal force (RCF) of 1700 × g, duration 10 min). The plasma was transferred into amber cryovials and stored in a −80 °C freezer. Special attention must be paid to the time interval from blood collection to plasma separation and storage at either − 20 °C or directly at −80 °C, which should be strictly controlled within 100 min (not exceeding 120 min). If temporarily stored at −20 °C, samples must be transferred to a −80 °C freezer within 24 h. For long-term preservation, samples should be maintained at −80 °C.

### Determination of drug concentrations

Using stable isotope-labeled valsartan-D9 and amlodipine-D4 as internal standards, the concentrations of valsartan and amlodipine in healthy volunteers after oral administration of valsartan/amlodipine tablets were determined by a validated LC-MS/MS method^19^. Chromatographic separation was performed on a Welch Ultimate^®^ PFP column (2.1 × 50 mm, 5 μm) with mobile phase A consisting of 0.1% formic acid and 5 mM ammonium acetate in water, and mobile phase B consisting of 0.1% formic acid and 5 mM ammonium acetate in methanol. The injection volume was 8.0 µL, the flow rate was 0.6 mL/min, and the column temperature was maintained at 40 °C under gradient elution conditions. Data acquisition and analysis were conducted using Analyst 1.6.3 software.

### Pharmacokinetic parameters and statistical analyses

**Fig. 1:**
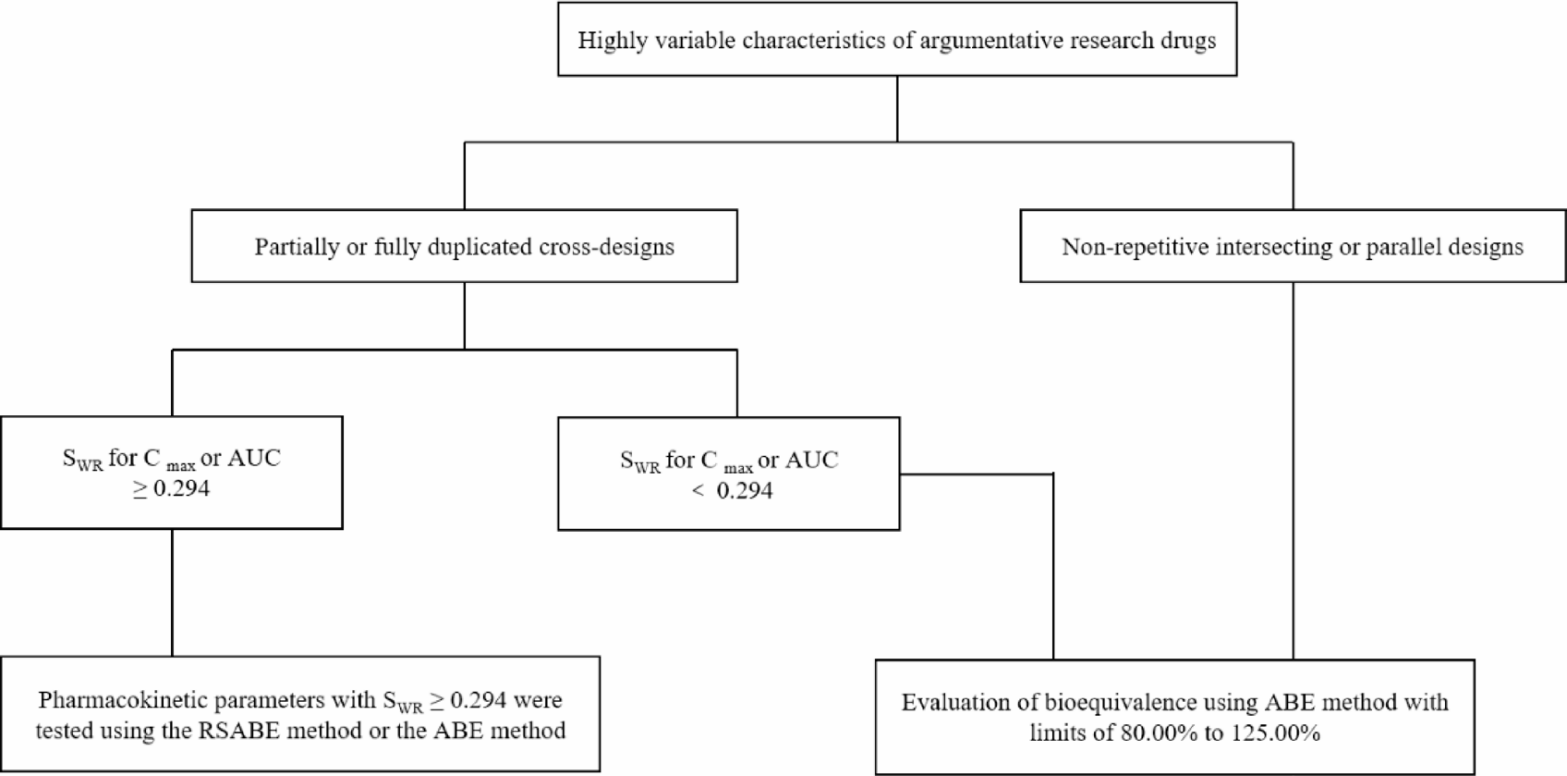
Decision tree for bioequivalence studies of highly variable drugs^20^.

The plasma concentration-time data of valsartan and amlodipine were processed using the Phoenix WinNonlin 7.0 software. The primary pharmacokinetic parameters included: AUC_0 − t_ (area under the concentration-time curve from dosing to a specific time point, t)、AUC_0−∞_ (area under the curve extrapolated to infinite time)、C_max_ (maximum concentration)、T_max_ (time to reach C_max_)、t_1/2_ (terminal elimination half-life)、λ_z_ (terminal elimina- tion rate constant)、AUC__% Extrap_ (percentage of AUC extrapolated). Bioequivalence evaluation was performed using either ABE or RSABE method, based on the within-subject standard deviation (S_WR_) of the PK parameters (AUC_0 − t_、AUC_0−∞_ and C_max_) for the reference drug of valsartan and amlodipine. The statistical analysis procedure and decision-making flowchart are presented in Fig. 1.

When S_WR_ is < 0.294, the ABE statistical method is applied for evaluation. The acceptance criterion is as follows: if the 90% confidence intervals (CIs) of the geometric mean ratios (GMRs) for AUC_0 − t_、AUC_0−∞_ and C_max_ between the test and reference drugs fall within the range of 80.00%−125.00%, bioequivalence between the test and reference drugs can be concluded.

The RSABE method is applied to the BE evaluation if S_WR_ ≥0.294 (CV_WR_% ≥30%) for any primary PK parameter. In this case, the 95% upper confidence bound for $(\bar{Y}_T-\bar{Y}_T)^2-\theta S_{WR}^2$ is calculated based on Howe’s Approximation I, where ${\overline {Y} _T}$ and ${\overline {Y} _R}$ are the natural log-transformed AUC or C_max_ mean values for the test and reference formulations, respectively. $\theta ={\left[ {\frac{{\ln 1.25}}{{\sigma \omega 0}}} \right]^2}$represent the threshold of the corrected bioequivalence and σw0 = 0.25 is the regulatory constant set by the US FDA and China NMPA. For any given primary PK parameter (s), bioequivalence is concluded if the 95% upper confidence bound (Critbound) for$(\bar{Y}_T-\bar{Y}_T)^2-\theta S_{WR}^2$ is ≤ 0 and the test/reference GMR (point estimate) falls within 80.00%–125.00%.

Bioequivalence between the test and reference drugs can only be concluded if all three parameters (C_max_、AUC_0 − t_ and AUC_0−∞_) are judged to be bioequivalent.

### Safety assessments

Safety evaluation indicators included physical examinations, vital sign measurements, electrocardiograms (ECGs), and laboratory tests conducted during the trial. The investigators monitored all adverse events (AEs) occurring in subjects throughout the clinical study, including abnormal clinical symptoms and medical examination findings. The severity of AEs in the study process was assessed by Common Terminology Criteria for Adverse Events 5.0 (CTCAE 5.0). The following parameters were documented: clinical manifestations, severity, onset time, resolution time, duration, interventions, and outcomes. Additionally, the causality between these events and the investigational drug was assessed.

## Result

### Study subjects’ demographics and dataset segmentation

According to the inclusion and exclusion criteria of the trial protocol, a total of 70 volunteers were screened under fed conditions, of whom 36 were enrolled and included in the Full Analysis Set (FAS). Among the 36 FAS volunteers, 26 were male and 10 were female. The mean age was 32.8 ± 5.8 years in the TRR group, 32.8 ± 7.2 years in the RTR group, and 32.3 ± 7.2 years in the RRT group. The mean BMI was 23.3 ± 1.9 kg/m² in the TRR group, 23.7 ± 2.1 kg/m² in the RTR group, and 23.2 ± 1.9 kg/m² in the RRT group.

Under fasting conditions, 103 volunteers were screened, with 36 enrolled and included in the FAS. Among these 36 FAS volunteers, 26 were male and 10 were female. The mean age was 30.1 ± 8.2 years in the TRR group, 32.2 ± 7.1 years in the RTR group, and 30.0 ± 8.9 years in the RRT group. The mean BMI was 23.0 ± 1.9 kg/m² in the TRR group, 22.1 ± 2.2 kg/m² in the RTR group, and 23.6 ± 1.8 kg/m² in the RRT group. All demographic and baseline characteristics of the volunteers are summarized in Table 2, demonstrating well-balanced distributions across groups.

**Table 2:** Demographic characteristics of all volunteers.

Demographic	Fed group (*N* = 36)			Fasting group(*N* = 36)		
	TRR (*N* = 12)	RTR (*N* = 12)	RRT (*N* = 12)	TRR (*N* = 12)	RTR (*N* = 12)	RRT (*N* = 12)
Age, year, Mean (SD)	32.8 (5.8)	32.8 (7.2)	32.3 (7.2)	30.1 (8.2)	32.2 (7.1)	30.0 (8.9)
Sex, n (%)						
Male	10 (83.3)	9 (75.0)	7 (58.3)	7 (58.3)	10 (83.3)	9 (75.0)
Female	2 (16.7)	3 (25.0)	5 (41.7)	5 (41.7)	2 (16.7)	3 (25.0)
Race, n (%)						
Han	12(100.0)	12 (100.0)	12 (100.0)	12 (100.0)	12 (100.0)	12 (100.0)
Height, (cm), Mean (SD)	168.3 (5.4)	170.0 (8.5)	166.6 (10.2)	167.8 (9.1)	167.8 (8.2)	167.8 (6.7)
Body weight, (kg), Mean (SD)	66.0 (7.0)	68.6 (8.9)	65.0 (11.5)	65.0 (8.4)	62.3 (8.6)	66.7 (7.6)
BMI, (kg/m^2^), Mean (SD)	23.3 (1.9)	23.7 (2.1)	23.2 (1.9)	23.0 (1.9)	22.1 (2.2)	23.6 (1.8)

*BMI* body mass index, *SD* standard deviation.

**Fig. 2:**
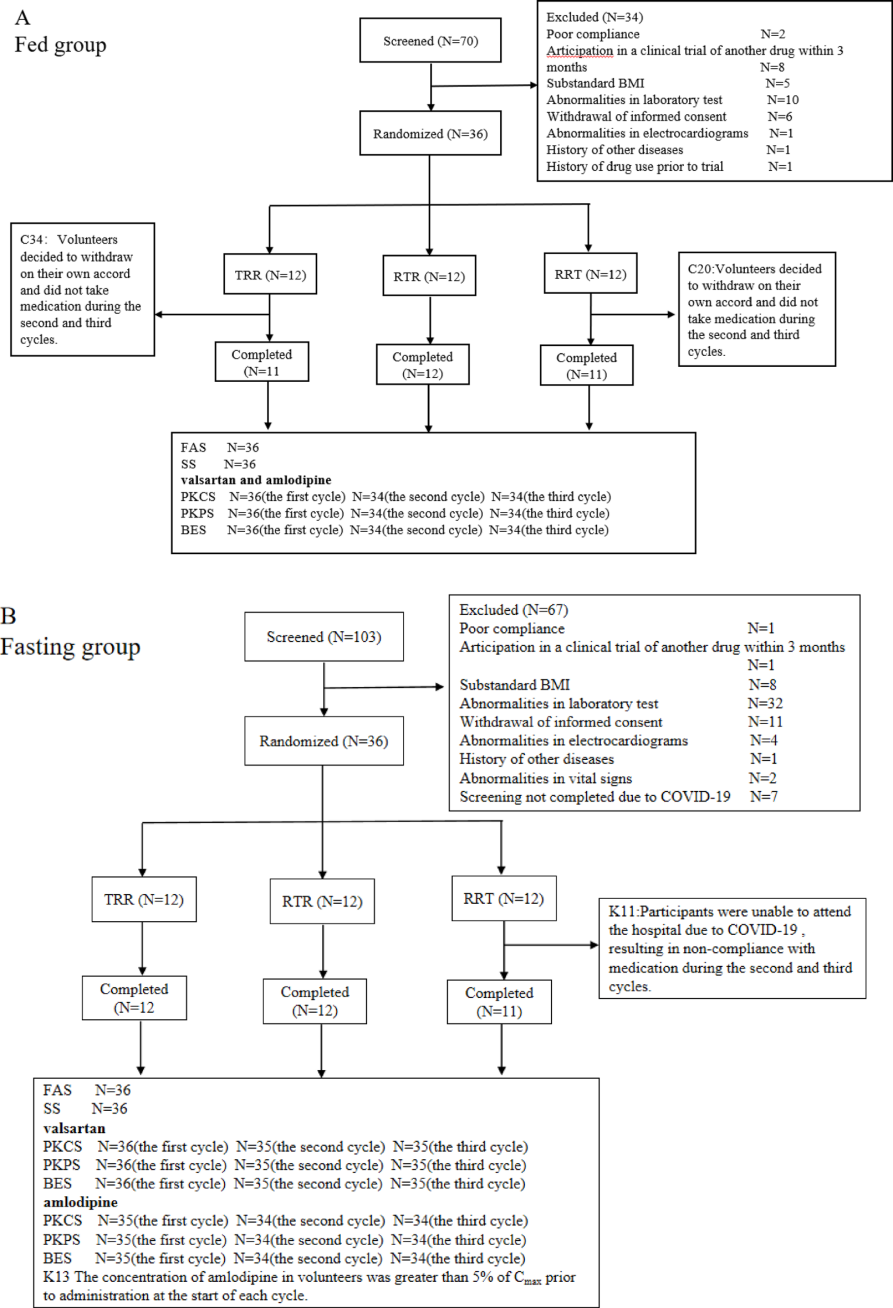
Reasons for volunteer screening failure, group characteristics, trial completion status, withdrawal status, and dataset division in this study (A: fed group; B: fasting group).

Figure 2 (A: fed group; B: fasting group) summarizes the reasons for screening failure, demographic characteristics, trial completion status, discontinuations, and dataset allocations among the study volunteers. In the fed trial, volunteers C20 and C34 voluntarily withdrew after completing the first cycle; thus, only 34 volunteers completed the study. A total of 36 volunteers were included in the full analysis set (FAS) and safety set (SS). For the PK concentration set (PKCS), PK parameter set (PKPS), and bioequivalence (BE) set (BES), 36 volunteers were included in the first cycle, 34 in the second cycle, and 34 in the third cycle.

In the fasting trial, 35 volunteers completed the study (volunteer K11 withdrew during the washout period of the first cycle due to COVID-19 (Corona Virus Disease 2019) pandemic-related lockdown restrictions preventing hospital visits). All 36 volunteers were included in the FAS and SS. For valsartan, 36 volunteers were included in the PKCS, PKPS, and BES in the first cycle, while 35 were included in the second and third cycles. For amlodipine, volunteer K13 exhibited pre-dose concentrations exceeding 5% of C_max_ in each cycle; therefore, 35 volunteers were included in the PKPS and BES in the first cycle, and 34 in the second and third cycles.

### Pharmacokinetic assessments

**Fig. 3:**
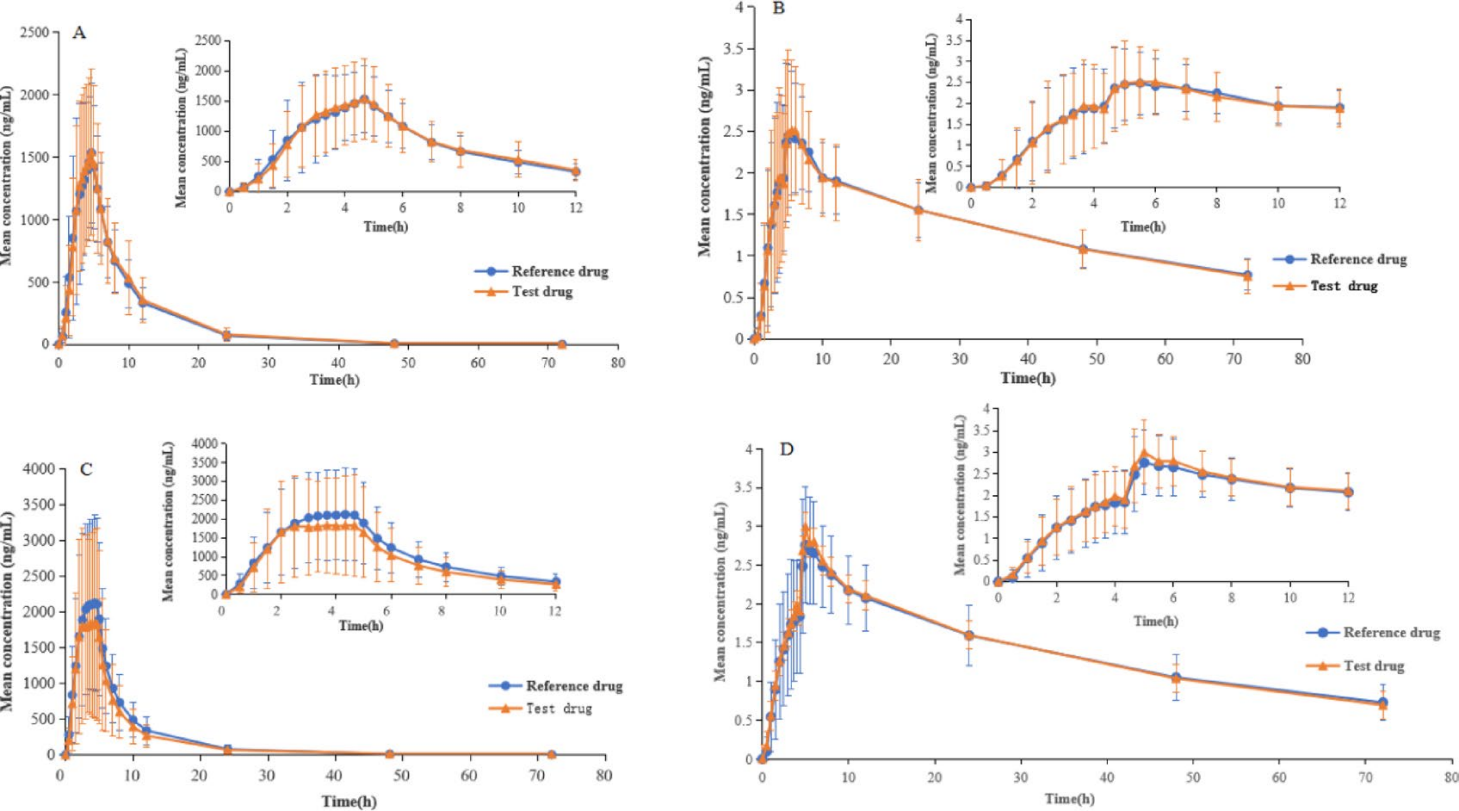
Mean (± standard error) plasma concentration-time curves of valsartan (A/C) and amlodipine (B/D) after a single oral administration of Valsartan/Amlodipine Tablets(I) under fed and fasting conditions (A/B: in the fed condition, C/D: in the fasting condition).

The mean plasma concentration-time curves of valsartan and amlodipine after oral administration of the test and reference drugs of Valsartan/Amlodipine Tablets(I) in volunteers under fed and fasting conditions are presented in Fig. 3. The pharmacokinetic parameters of valsartan and amlodipine following oral administration of the reference and test drugs under fed and fasting conditions are summarized in Table 3.

**Table 3:** Pharmacokinetic parameters of Valsartan/Amlodipine Tablets(I) following oral administration under fed and fasting conditions (Mean ± SD (CV%)).

Item	Parameter(units)	Fed group		Fasting group	
		Test(*N* = 36)	Reference(*N* = 36)	Test(*N* = 36)	Reference(*N* = 36)
Valsartan	C _max_ (ng/mL)	1,757.65 ± 555.33(31.60)	1,763.45 ± 565.39(32.06)	2,686.78 ± 1,231.88(45.85)	2,589.28 ± 1,258.49(48.60)
	AUC_0 − t_ (h*ng/mL)	12,633.35 ± 4,133.65(32.72)	12,152.43 ± 3,771.61(31.04)	15,922.83 ± 6,218.05(39.05)	16,303.08 ± 8,471.11(51.96)
	AUC_0−∞_(h*ng/mL)	13,034.42 ± 4,167.78(31.98)	12,563.07 ± 3,837.53(30.55)	16,357.81 ± 6,316.70(38.62)	16,767.00 ± 8,588.86(51.22)
	^*^T_max_ (h)	4.00(1.50、10.00)	4.33(1.50、8.01)	4.33(1.50、7.00)	3.33(1.00、5.00)
	t_1/2_ (h)	5.68 ± 1.93(33.93)	5.72 ± 2.07(36.17)	5.57 ± 1.79(32.23)	6.54 ± 2.89(44.18)
	λ_z_	0.13 ± 0.03(23.79)	0.13 ± 0.03(24.71)	0.13 ± 0.03(24.49)	0.12 ± 0.04(31.39)
^**^Amlodipine	C _max_ (ng/mL)	2.86 ± 0.78(27.16)	2.81 ± 0.72(25.80)	3.19 ± 0.62(19.53)	3.04 ± 0.68(22.21)
	AUC_0 − t_ (h*ng/mL)	95.46 ± 21.24(22.25)	95.97 ± 18.92(19.72)	98.40 ± 20.09(20.42)	98.09 ± 21.40(21.81)
	AUC_0−∞_(h*ng/mL)	150.12 ± 40.87(27.22)	153.11 ± 38.14(24.91)	140.65 ± 35.76(25.43)	144.62 ± 39.42(27.25)
	^*^T_max_ (h)	5.00(2.50、23.99)	5.50(2.00、24.00)	5.00(4.66、12.00)	5.00(3.00、12.02)
	t_1/2_ (h)	48.21 ± 11.85(24.58)	49.69 ± 14.31(28.80)	40.48 ± 9.44(23.32)	42.57 ± 9.15(21.50)
	λ_z_	0.02 ± 0.00(21.11)	0.01 ± 0.00(24.56)	0.02 ± 0.00(21.26)	0.02 ± 0.00(23.12)

^*^Data are presented as the arithmetic mean ± standard deviation, except for T_max_, which is represented as median (minimum-maximum). ^**^ Amlodipine *N* = 36(Fed group) *N* = 35(Fasting group).

For the reference drug of valsartan in fed condition, the S_WR_ values for C_max_、AUC_0 − t_ and AUC_0−∞_ were all less than 0.294. Therefore, ABE was employed for assessment. The GMRs (test/reference) and their 90% CIs for C_max_、AUC_0 − t_ and AUC_0−∞_ were 99.86%, 103.84%, and 103.64%, respectively, with 90% CIs of 92.29%–108.04%, 97.55%–110.54%, and 97.57%–110.10%, respectively. All values fell within the predefined bioequivalence range of 80.00%–125.00%, meeting the acceptance criteria for bioequivalence. Similarly, the S_WR_ values for C_max_、AUC_0 − t_ and AUC_0−∞_ of amlodipine were also less than 0.294, and the 90% confidence intervals were all within the bioequivalence criteria range, meeting the acceptance criteria for bioequivalence. This indicates that the test drug and reference drug are bioequivalent under postprandial administration conditions.

Under fasting conditions, the S_WR_ values for C_max_、AUC_0 − t_ and AUC_0−∞_ for valsartan all exceeded 0.294, necessitating evaluation by the RSABE approach. In contrast, the S_WR_ values for amlodipine’s C_max_、AUC_0 − t_ and AUC_0−∞_ were all below 0.294, requiring assessment via ABE method. Notably, the point estimates of the GMRs (test/reference) for valsartan’s C_max_、AUC_0 − t_ and AUC_0−∞_ all fell within the 80.00%–125.00% range, with the upper limits of the one-sided 95% CIs all below 0. Similarly, the 90% CIs of the GMRs for amlodipine’s C_max_、AUC_0 − t_ and AUC_0−∞_ were entirely contained within the 80.00%–125.00% range. These results confirm that both formulations meet the predefined bioequivalence acceptance criteria, demonstrating that the test and reference drugs are bioequivalent under fasting conditions.

Plasma drug concentration analysis revealed the following C_max_ values: in the fed group, valsartan (test drug: 1,757.65 ng/mL, reference drug: 1,763.45 ng/mL) and amlodipine (test drug: 2.86 ng/mL, reference drug: 2.81 ng/mL); in the fasting group, valsartan (test drug: 2,686.78 ng/mL, reference drug: 2,589.28 ng/mL) and amlodipine (test drug: 3.19 ng/mL, reference drug: 3.04 ng/mL). No statistically significant differences were observed between the test and reference formulations. Compared with the fasting group, the C_max_ values of both valsartan and amlodipine were reduced in the fed group, potentially attributable to physicochemical interactions between the drugs and food components that may affect drug absorption following oral administration.

### Bioequivalence analysis

The bioequivalence analysis was performed using Phoenix WinNonlin 7.0 software, employing both ABE and RSABE method. The statistical results are presented in Table 4.

**Table 4:** Average bioequivalence statistical results of Valsartan/Amlodipine Tablets(I) reference Preparation scale.

Group	Component	PK Parameters (unit)	ABE		RSABE			CV (%)
			Ratio(T/R) (%)	90%CI	S_*WR*_ [> = 0.294]	Point Estimate [0.80,1.25]	Critical Bound [< = 0]	
Fed group	Valsartan (*N* = 36)	C _max_ (ng/mL)	99.86	92.29%~108.04%	0.232	0.9986	−0.0292	23.57
		AUC_0 − t_ (h*ng/mL)	103.84	97.55%~110.54%	0.204	1.0379	−0.0188	20.12
		AUC_0−∞_(h*ng/mL)	103.64	97.57%~110.10%	0.199	1.0359	−0.0181	19.61
	Amlodipine (*N* = 36)	C _max_ (ng/mL)	101.34	93.63%~109.70%	0.188	1.0184	−0.0154	18.55
		AUC_0 − t_ (h*ng/mL)	99.34	95.59%~103.24%	0.098	0.9939	−0.0046	9.59
		AUC_0−∞_(h*ng/mL)	98.45	93.68%~103.46%	0.111	0.9788	−0.0037	10.94
Fasting group	Valsartan (*N* = 36)	C _max_ (ng/mL)	104.21	92.12%~117.87%	0.4	1.0496	−0.0794	40.98
		AUC_0 − t_ (h*ng/mL)	102.12	93.19%~111.90%	0.306	1.0281	−0.0487	31.25
		AUC_0−∞_(h*ng/mL)	101.82	93.12%~111.34%	0.301	1.0246	−0.0477	30.71
	Amlodipine (*N* = 35)	C _max_ (ng/mL)	105.67	100.16%~111.49%	0.139	1.0561	−0.0024	14.11
		AUC_0 − t_ (h*ng/mL)	101.01	97.95%~104.17%	0.091	1.0098	−0.004	9.18
		AUC_0−∞_(h*ng/mL)	97.99	94.68%~101.41%	0.115	0.9793	−0.0059	11.49

### Safety analysis

In the fed group, 36 volunteers were included in the Safety Set (SS). A total of 16 volunteers experienced 17 AEs (3 AE cases with drug T and 14 with drug R), yielding an AE incidence rate of 44.4% (16/36). Among these, 12 AEs were mild in severity, while 5 were moderate. Outcomes of AEs: Drug T: Recovery rate: 66.7% (2/3 cases); Lost to follow-up: 33.3% (1/3 cases). Drug R: Recovery rate: 64.3% (9/14 cases); Improvement: 7.1% (1/14 cases); Lost to follow-up: 28.6% (4/14 cases).

In the fasting group, 36 volunteers were included in the SS. A total of 22 volunteers experienced 31 AEs (14 cases/drug T and 17cases/drug R), with an AE incidence rate of 61.1% (22/36). Among these, 30 AEs were mild in severity, while 1 was moderate. Notably, abnormal urinalysis findings primarily involved increased urinary mucus in routine urine tests. Outcomes of AEs: Drug T: Recovery rate: 92.9% (13/14 cases); Lost to follow-up: 7.1% (1/14 cases). Drug R: Recovery rate: 82.4% (14/17 cases); Improvement: 11.8% (2/17 cases); Lost to follow-up: 5.9% (1/17 cases). All adverse medical events observed in volunteers following administration of the investigational drugs are summarized in Table 5, which details the system-wise AE profiles of both the test and reference drugs.

**Table 5:** AEs of Valsartan/Amlodipine Tablets(I) under fasting or fed studies in healthy Chinese volunteers.

Classification of system organs (SOC)	Fed group				Fasting group			
	Test drug (*N* = 35)		Reference drug (*N* = 35)		Test drug (*N* = 35)		Reference drug (*N* = 36)	
Preferred terminology (PT)	case	Rate(%)	case	Rate(%)	case	Rate(%)	case	Rate(%)
Metabolic and nutritional diseases								
TG (Triglycerides) elevated	1	2.9	11	31.4	2	5.7	2	5.6
Various inspections								
Serum creatine phosphokinase decreased	1	2.9	1	2.9	3	8.6	5	13.9
Abnormal urinalysis	0	0	0	0	3	8.6	4	11.1
Urine leukocytes positive	1	2.9	1	2.9	2	5.7	1	2.8
Urine erythrocyte positive	0	0	0	0	1	2.9	1	2.8
Heart rate elevated	0	0	0	0	1	2.9	1	2.8
Blood creatinine decreased	0	0	0	0	0	0	1	2.8
Blood pressure decreased	0	0	1	2.9	0	0	0	0
**Systemic diseases and various reactions at the site of administration**								
Fever	0	0	0	0	2	5.7	2	5.6

## Discussion

This study evaluated the bioequivalence of two Valsartan-Amlodipine Tablets (I) in healthy Chinese volunteers to support the clinical application of generic formulations. Furthermore, we assessed the PK data between the fasting and fed trials and found that food did not influence the AUC_0–∞_ and C_max_ values of amlodipine for either the T or R formulations. However, the main PK parameters (C_max_, AUC_0–t_, and AUC_0–∞_) of valsartan in the fed condition were significantly decreased compared with that in the fasting condition. We hypothesize that due to structural and solubility differences between amlodipine and valsartan, the pharmacokinetic parameters of the more lipophilic valsartan are susceptible to high-fat dietary effects, whereas amlodipine remains unaffected. Valsartan exhibits poor dissociation and readily absorbs under physiological pH conditions. Typically, food elevates gastric pH and slows gastric emptying rate, leading to increased valsartan dissociation and consequently reduced drug absorption^21^. Furthermore, physical or chemical interactions between drugs and food may affect drug absorption following oral administration. Our study demonstrated that the rate and extent of absorption of valsartan were significantly reduced under fed conditions, whereas no such effect was observed for amlodipine. Multiple factors may contribute to this discrepancy, including ethnicity, inter-individual variability, and metabolic enzyme genotypes. Genetic polymorphisms in metabolic enzyme genes can alter the metabolic activity toward various clinically important drugs^22^. However, based on current evidence, it remains difficult to infer the specific contributing factors.

In this study, AEs occurring during the trial period were collected and evaluated. All reported AEs were of mild to moderate severity with favorable recovery, and no serious adverse events (SAEs) were observed. In the fed condition, 17 AE cases were reported, of which 5 cases (2 cases/drug T and 3 cases/drug R) were assessed as possibly drug-related, while the remaining 12 cases were deemed unlikely to be drug-related. In the fasting conditions, 31 AE cases occurred, with 21 cases (9 cases/drug T and 12 cases/drug R) classified as possibly drug-related, 6 cases as definitely drug-unrelated, and 4 cases as unlikely to be drug-related. The incidence of AEs in the fed condition was lower than that in the fasting condition, and the test drug demonstrated a lower AE incidence compared to the reference drug.This outcome may be attributed to the significantly lower C_max_ of valsartan after meals compared to fasting conditions, coupled with the test formulation exhibiting a lower C_max_ than the reference formulation. These results indicate that the generic drug maintains an acceptable safety profile for Chinese volunteers participating in the study.

Finally, the study data are compared with previously conducted studies in other countries and regions, and these data are presented in Table 6. The C_max_ and AUC_0 − t_ (h·ng/mL) of valsartan under both fed and fasting conditions were similar to previously published data collected from healthy Chinese subjects but slightly lower than those reported in studies involving non-Chinese populations. These discrepancies may be attributed to differences in volunteer cohorts, administered doses, and reagents used across studies. Assuming that the impact of dosage on the trial is limited to simple dose superposition, the C_max_, T_max_ (h), t_1/2_, and AUC_0 − t_ of amlodipine under fed and fasting conditions were comparable to previously reported data. This suggests that differences in volunteer populations (e.g., Chinese, American, Korean) have minimal influence on the systemic exposure of amlodipine. These findings further indicate that, compared with amlodipine, valsartan exhibits a higher intrasubject coefficient of variation.

**Table 6:** Comparison of PK parameters in this study with previous published studies.

Source			*N*	Dosage	Region	C_max_ (ng/mL)	^*^T_max_ (h)	t_1/2_ (h)	AUC_0 − t_ (h*ng/mL)
Fed group	Valsartan	Test drug in this study	36	80 mg/5 mg	China	1,757.65 ± 555.33	4.00(1.50、10.00)	5.68 ± 1.93	12,633.35 ± 4,133.65
		Reference drug in this study	36	80 mg/5 mg	China	1,763.45 ± 565.39	4.33(1.50、8.01)	5.72 ± 2.07	12,152.43 ± 3,771.61
		Previous study ([8])	51	80 mg/5 mg	China	1,520 ± 537	3.23 ± 1.49	8.51 ± 2.37	10377.61 ± 3,416.53
		Previous study ([5])	44	80 mg/5 mg	China	1,364.56 ± 534.53	4.49(0.99, 5.99)	5.46 ± 1.96	8,329.16 ± 2,911.29
		Previous study ([9])	17	160 mg	India	5,940 (2,370)	2.90 (1.07)	4.31 (0.99)	34,520(15,740)
		Previous study ([23])	33	160 mg/10 mg	United States	3,497 ± 1671	4 (1–10)	10.5 ± 6.8 (65)	22,943 ± 10,199 (44.5)
	Amlodipine	Test drug in this study	36	80 mg/5 mg	China	2.86 ± 0.78	5.00(2.50、23.99)	48.21 ± 11.85(24.58)	95.46 ± 21.24
		Reference drug in this study	36	80 mg/5 mg	China	2.81 ± 0.72	5.50(2.00、24.00)	49.69 ± 14.31(28.80)	95.97 ± 18.92
		Previous study ([8])	51	80 mg/5 mg	China	2.62 ± 0.593	5.50 ± 2.78	36.84 ± 8.13	94.96 ± 17.49
		Previous study ([5])	44	80 mg/5 mg	China	3.67 ± 0.86	5.49(1.99, 10.99)	44.26 ± 11.30	119.65 ± 28.57
		Previous study ([23])	33	160 mg/10 mg	United States	4.8 ± 1.3	8 (6–24)	45.4 ± 9.2 (20)	243 ± 79 (27)
Fasting group	Valsartan	Test drug in this study	36	80 mg/5 mg	China	2,686.78 ± 1,231.88	4.33(1.50、7.00)	5.57 ± 1.79	15,922.83 ± 6,218.05
		Reference drug in this study	36	80 mg/5 mg	China	2,589.28 ± 1,258.49	3.33(1.00、5.00)	6.54 ± 2.89	16,303.08 ± 8,471.11
		Previous study ([8])	53	80 mg/5 mg	China	2,960 ± 1,350	3.34 ± 0.86	9.33 ± 3.81	17,552.47 ± 7,523.72
		Previous study ([5])	44	80 mg/5 mg	China	2,799.64 ± 1,385.07	2.99(0.99, 6.99)	5.58 ± 1.78	15,873.30 ± 6,751.62
		Previous study ([23])	33	160 mg/10 mg	United States	4,138 ± 1,993	2.5 (1–4)	10.2 ± 6.4 (63)	24,211 ± 10,522 (43.5)
		Previous study ([24])	24	160 mg/10 mg	South Korea	3,796.73	2.92	6.56	25432.01
	Amlodipine	Test drug in this study	35	80 mg/5 mg	China	3.19 ± 0.62	5.00(4.66、12.00)	40.48 ± 9.44	98.40 ± 20.09
		Reference drug in this study	35	80 mg/5 mg	China	3.04 ± 0.68	5.00(3.00、12.02)	42.57 ± 9.15	98.09 ± 21.40
		Previous study ([8])	53	80 mg/5 mg	China	2.51 ± 0.553	6.35 ± 1.63	37.17 ± 8.97	92.08 ± 20.12
		Previous study ([5])	44	80 mg/5 mg	China	3.94 ± 0.92	4.99(4.49, 12.01)	41.77 ± 11.08	120.44 ± 27.58
		Previous study ([23])	33	160 mg/10 mg	United States	4.6 ± 1.3	8 (6–12)	46.3 ± 11.2 (24)	223 ± 85 (38)
		Previous study ([24])	24	160 mg/10 mg	South Korea	5.92	6.42	40.96	255.98

Similar to most bioequivalence studies, this study also has certain limitations. First, the study subjects were a small number of healthy young volunteers who met strict inclusion and exclusion criteria. Due to disease status, comorbidities, or drug interactions, the pharmacokinetic parameters in healthy volunteers may differ from those in actual patients and may not represent a broader population (e.g., patients, elderly individuals, children, or other ethnic groups), thereby limiting clinical relevance. It is necessary to conduct further studies in larger populations, including target patients and elderly individuals. Furthermore, the single-dose design and short-term follow-up cannot assess adverse events (AEs) associated with long-term medication use. Given that the primary objective of this study was to evaluate the pharmacokinetic (PK) comparability between the test drug and the reference drug, long-term administration in healthy volunteers lacks sufficient clinical justification. For further safety assessment, longitudinal follow-up studies should be implemented.

The current results show that valsartan is an highly variable drug (HVD), and that Valsartan/Amlodipine Tablets (I) represents a complex problem in terms of BE assessment. A replicate crossover study can be conducted if an applicant suspects that the absorption rate and/or extent of a drug product are highly variable. The main purpose of our study is to support an increase in the supply of Valsartan/Amlodipine Tablets (I) in China by proving the efficacy and safety of a generic alternative. For bioequivalence evaluation, we used two NMPA-specified methods (ABE and RSABE) in this study which can be used as a reference for the design and implementation of future clinical trials.

## Conclusion

The Valsartan/Amlodipine Tablets (I) produced by Hubei Wushi Pharmaceutical Co., Ltd. were found to have mild adverse reactions and good safety after single-dose oral administration to Chinese healthy volunteers, and were bioequivalent to the reference drug, Exforge^®^. They can be substituted for each other in clinical practice. The results of this study provide support for the market launch of the new generic valsartan/amlodipine tablets in China.

On the other hand, the Valsartan/Amlodipine Tablets (I), as a representative antihypertensive fixed-dose combination (FDC), has established a template for BE studies of other FDCs through its “component-specific differential statistical strategy.” This approach effectively addresses the statistical challenges associated with HVDs. These methodological insights can be directly applied to clinical trials of similar drug products, thereby enhancing success rates while mitigating development risks.

## Data Availability

Data may be obtained from the corresponding authors upon reasonable request, but all applicable legal requirements must still be complied with in order to protect the confidentiality of the personal information of research participants.
